# Jottings

**Published:** 1909-11-27

**Authors:** 


### Jottings.

The Mayor of Stafford has given £100 to the Staffordshire General Infirmary, and has started a million penny fund in aid of the institution.

A new terrazzo-mosaic floor, the gift of the United Friendly Societies Council, has been laid down in the operating theatre and anæsthetising room of the Shropshire Infirmary.

In response to the directors' appeal the sum of £11,328 8s. 5d. has been received or promised towards the Building and Improvement Fund for the Salop Infirmary New Nurses' Home and Mortuary.

A new five-story hospital, to be known as St. Joseph's Hospital, is being constructed in Vancouver, Washington, at the cost of $125,000. The building will be 136 ft. by 48 ft. and 76 ft. high. There will be 128 rooms, and also a solarium at each end.

The American Hospital, built and equipped through the generosity of the American colony in Paris, which was formally opened on October 28 last, is beautifully situated at Neuilly, and is surrounded by spacious grounds. It contains twenty-five beds, most of which have already been endowed.

The *Building News* publishes plans of a new hydro to be erected at Harrogate on a site which embraces an area of about six acres. Provision has been made for the usual reception-rooms, about 300 bedrooms, and a complete suite of baths, together with garage, stabling, etc. The laying-out of the ornamental grounds has been designed by Messrs. J. Cheal and Son, of Crawley and London. The architect is Mr. Samuel Stead, who at one time held the post of borough engineer.

The Bishop of Stepney will open the new chapel of the Metropolitan Hospital on December 1.

Last week the Mayoress of Milton laid the foundation stone of the new Servants' Home at the local Municipal Infectious Diseases Hospital.

At a well-attended public meeting held last week at Loughton it was decided to raise funds to provide and equip a hospital to satisfy the medical and surgical needs of Buck-

At the Derbyshire Hospital for Sick Children a brass plate has been fixed on one of the cots bearing the following inscription: "Supported by the children in some of the schools under the Derbyshire County Council."

hurst Hill, Loughton, and the surrounding district.

The County Committee has accepted Lord Forester's offer of a site on his estate, a mile from Willey, for the proposed sanatorium for Salop. The position is an elevated one in the heart of a pine forest.

The Medical Officer of Dalkeith burgh reports that the Fever Hospital of the borough is inadequate to deal with more than one infectious disease at a time because of the insufficient nursing staff and of the want of sufficient accommodation. The hospital, he declares, must be reconstructed entirely, and at least two separate pavilions and one observation ward are necessary.

A Local Government Board inquiry was recently held into an application made by the King's Norton and Northfield Urban District Council for sanction to borrow £200 for the purpose of erecting observation cubicles at the Isolation Hospital, for the purpose of treating cases of mixed infection or of doubtful diagnosis. There was no opposition to the scheme.

